# Supplementary figures and images for: Seed Germination after 30 Years Storage in Permafrost
Source: Plants (Basel). 2020 May 2;9(5):579. doi: 10.3390/plants9050579 (PMC7285074; doi:10.3390/plants9050579)

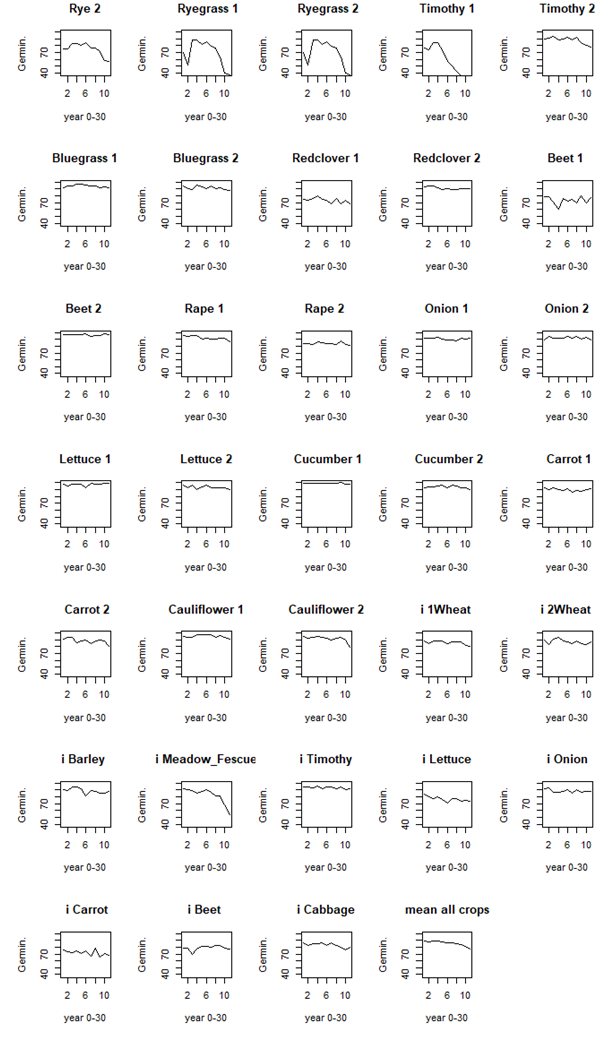

Supplement: Supplementary file 1 [file plants-09-00579-s001.tif]
